# ASK1 is involved in cognitive impairment caused by long-term high-fat diet feeding in mice

**DOI:** 10.1038/srep10844

**Published:** 2015-06-05

**Authors:** Kensuke Toyama, Nobutaka Koibuchi, Yu Hasegawa, Ken Uekawa, Osamu Yasuda, Daisuke Sueta, Takashi Nakagawa, Mingjie Ma, Hiroaki Kusaka, Bowen Lin, Hisao Ogawa, Hidenori Ichijo, Shokei Kim-Mitsuyama

**Affiliations:** 1 Department of Pharmacology and Molecular Therapeutics, Kumamoto University Graduate School of Medical Sciences, Kumamoto; 2 Department of Cardiovascular Medicine, Kumamoto University Graduate School of Medical Sciences, Kumamoto; 3 Department of Cardiovascular Clinical and Translational Research, Kumamoto University Hospital, Kumamoto; 4 Laboratory of Cell Signaling, Graduate School of Pharmaceutical Sciences, The University of Tokyo, Japan

## Abstract

Although high-fat diet intake is known to cause obesity and diabetes, the effect of high-fat diet itself on cognitive function remains to be clarified. We have previously shown that apoptosis signal-regulating kinase 1 (ASK1) is responsible for cognitive impairment caused by chronic cerebral hypoperfusion. The present work, by using ASK1 deficient mice, was undertaken to explore the influence of chronic high-fat diet intake on cognitive function and the role of ASK1. Cognitive function in wild-type mice fed high-fat diet from 2 to 24 months of age was significantly impaired compared to those fed control diet, which was associated with the significant white matter lesions, reduction of hippocampal capillary density, and decrement of hippocampal neuronal cell. However, ASK1 deficiency abolished the development of cognitive impairment and cerebral injury caused by high-fat diet. Our results provided the evidence that high-fat diet itself causes cognitive impairment and ASK1 participates in such cognitive impairment.

Dementia cases is increasing as the population ages, causing the financial cost of dementia to increase in developed countries^1^. Alzheimer’s disease is the primary form of dementia and vascular dementia is the second most common cause of dementia after Alzheimer’s disease. Vascular dementia and Alzheimer’s disease are caused by the common vascular risk factors, possibly leading to a vascular drainage failure of β-amyloid, and cerebrocapillary damage caused by cerebral hypoperfusion^2^,^3^,^4^,^5^. However, despite the high level of need, at present, no sufficient therapeutic strategy for dementia is available.

Obesity, the main component of metabolic syndrome, is associated with insulin resistance, diabetes, and hypertension, which are vascular risk factors responsible for the pathogenesis of cognitive decline^6^,^7^. It is well established that chronic high-fat (high cholesterol) diet intake is one of the causes of obesity, insulin resistance, and glucose intolerance^8^,^9^,^10^. Although there is close relationship between metabolic syndrome and cognitive impairment^2^,^6^,^7^, the direct influence of long-term high fat diet intake on cognitive function remains to be clarified.

Apoptosis signal-regulating kinase 1 (ASK1)^11^, a member of the mitogen-activated protein kinase family, plays a key role in cellular responses induced by a variety of stressors including oxidative stress and inflammation^12^,^13^,^14^. By using ASK1 deficient mice, we have previously shown that ASK1 is involved in the impairment of cognitive function caused by chronic cerebral hypoperfusion with partial stenosis of bilateral common carotid artery^15^. Therefore, the present study was performed to examine whether long-term high-fat diet feeding can cause cognitive impairment in mice, and if so, to examine whether ASK1 can be implicated in cognitive decline caused by high-fat diet feeding.

## Results

### Body weight, adipose weight, muscle weight, and survival rate of long-term high-fat diet fed mice

**Figure 1:**
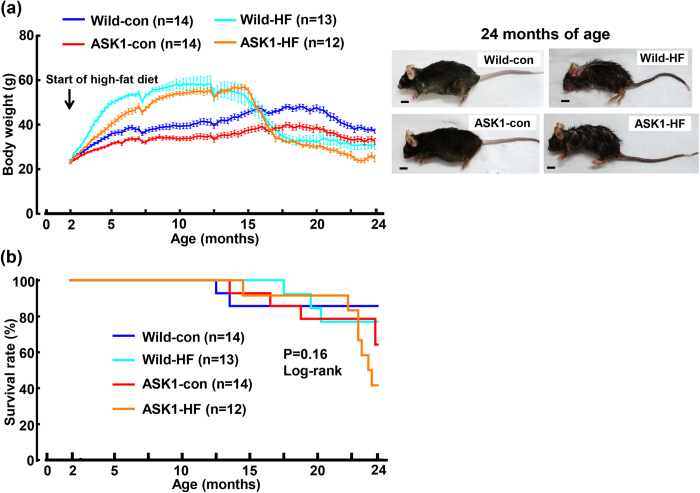
Effects of long-term high-fat diet feeding on body weight (**a**) and survival rate (**b**) of wild-type mice and ASK1-/- mice. Left panel in (**a**) shows the change in body weight of each group of mice from 2 months to 24 months of age. The right panels in (**a**) indicate the gross appearance of each group of 24-month-old mouse. Scale bar = 1 cm in (**a**). (**b**) indicates Kaplan-Meier curves of survival rate of each group of mice. Abbreviations used: Wild-con, wild-type mice fed control diet; Wild-HF, wild-type mice fed high-fat diet; ASK1-con, ASK1-/- mice fed control diet; ASK1-HF, ASK1-/- mice fed high-fat diet. Values are the means ± SEM. The number within parenthesis indicates the number of mice examined. Since mice were died during the study in Fig. 1(b), we could not perform two-way ANOVA among all groups in Fig. 1(a).

Figure 1(a) and Supplementary Figure 1 indicate time course of body weight in wild-type and ASK1-/- mice fed high-fat diet from 2 to 24 months of age (for 22 months), compared with those fed control diet. In both strains of mice, high-fat diet significantly and progressively increased body weight compared with control diet until 14 months of age. However, thereafter, the body weight of high-fat diet fed mice rapidly and significantly decreased compared with those fed control diet. Furthermore, body weight of ASK1-/- mice was smaller than that of wild-type mice, irrespective of high-fat or control diet, although there was no significant difference in the tibia length among all groups (Supplementary Figure 2(a)). As shown in Supplementary Figure 2(b) and (c), the change in body weight of both strains of mice was associated with the change in white adipose weight and adipose cell size. There was the significant correlation between body weight and white adipose weight (Supplementary Figure 2 (d)). Moreover, the reduction of body weight by high-fat diet was also associated with the reduction of skeletal muscle weight (Supplementary Figure 3 (a),(b)) and the reduction of muscle activity (Supplementary Figure 3 (c)). Compared with wild-type mice fed high-fat diet, skeletal muscle weight was smaller and muscle activity was less in ASK1-/- fed high-fat diet. The significant positive relationship between body weight and muscle weight was observed in 24-month-old mice (Supplementary Figure 3 (d) and (e)). On the other hand, no significant difference in food intake was noted between wild-type and ASK1-/- mice fed high-fat diet (Supplementary Figure 4).

As shown in Fig. 1(b), survival rate did not differ among 4 groups of mice (P = 0.16).

### Cognitive function of wild-type and ASK1-/- mice fed high-fat or control diet

**Figure 2:**
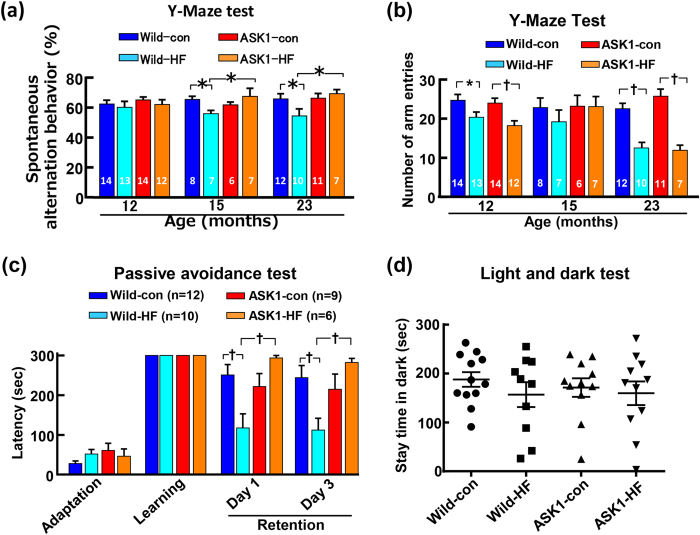
Effects of long-term high-fat diet feeding on cognitive function and anxiety. (**a**) and (**b**) indicate the spontaneous alteration behavior and number of arm entries, respectively, on the Y-maze test. (**c**) indicates latency of each group of 23-month-old mice on the passive avoidance test. (**d**) indicates the stay time in the dark side of 23-month-old mice on the Light and Dark test. Abbreviations used are the same as in Fig. 1. Values are the means ± SEM. The number within the bar in (**a**) and (**b**) indicates the number of mice examined. The number within parenthesis in (**c**) indicates the number of mice examined. * P < 0.05,†P < 0.01 between groups.

As shown by the Y-maze test in Fig. 2(a), spontaneous alteration behavior was significantly lower in wild-type mice fed high-fat diet than in those fed control diet at 15 and 23 months of age. On the other hand, spontaneous alteration behavior did not decrease in ASK1-/- mice fed high-fat diet compared with those fed control diet throughout the experiments. There was no significant correlation between spontaneous alteration behavior and body weight in 23-month-old mice (r = 0.042, p = 0.796) (Supplementary Figure 5 (a)) or 24-month-old white adipose weight(r = 0.130, p = 0.448) (Supplementary Figure 5 (b)). As shown in Fig. 2(b), there was no difference in number of arm entries between wild type and ASK1-/- mice throughout the experiment, regardless of high-fat or control diet. As shown by the passive avoidance test of 23-month-old mice in Fig. 2(c), the latency in high-fat diet-fed wild-type mice significantly decreased compared to that in control diet-fed wild-type mice, while that in ASK1-/- mice fed high-fat diet did not significantly decrease compared with ASK1-/- mice fed control diet. Figure 2(d) showed that high-fat diet did not affect stay time in dark (anxiety-like responses) in both strains of mice.

### White matter lesion, hippocampal capillary density and neuron number, cerebral mRNA levels, and amyloid β

**Figure 3:**
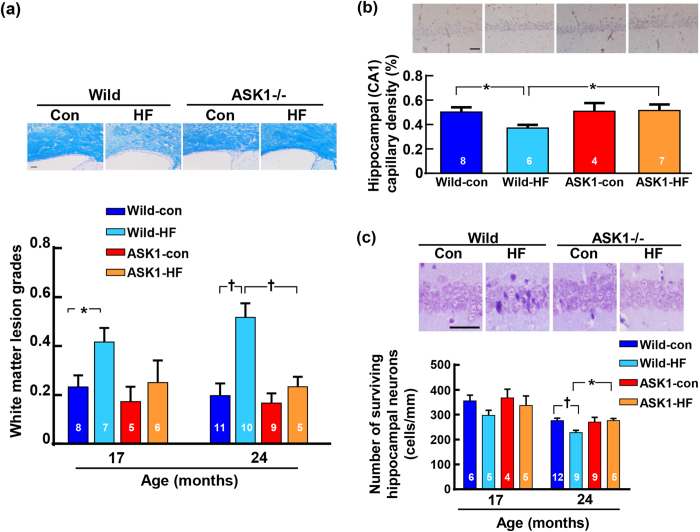
Effects of long-term high-fat diet feeding on cerebral white matter (**a**), hippocampal capillary density (**b**), and hippocampal neuron (**c**). In (a), upper panels indicate representative photomicrograph of Klüver-Barrera-stained sections of white matter corpus callosum from 24-month-old mice. In (**b**), upper panels indicate representative photomicrograph of hippocampal sections immunostained with anti-CD31 antibody from 17-month-old mice. In (**c**), upper panels indicate representative photomicrograph of Nissl-stained hippocampal (CA1) neuron from 17-month-old mice. Scale bar = 50 μm in (**a**), (**b**) and (**c**). Values are the means ± SEM. The number within the bar in (**a**), (**b**) and (**c**) indicates the number of mice examined. * P < 0.05,†P < 0.01 between groups.

High-fat diet feeding significantly enhanced white matter lesion in wild-type mice at 17 and 24 months of age while failed to enhance it in ASK1-/- mice (Fig. 3a). High-fat diet significantly reduced hippocampal capillary density and significantly decreased hippocampal neuronal cell number in wild-type mice, but not in ASK1-/- mice (Fig. 3b,c).

**Figure 4:**
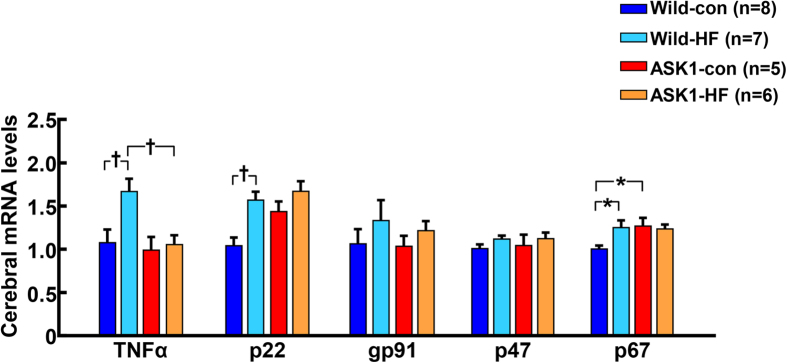
Effects of long-term high-fat diet feeding on cerebral mRNA levels for tumor necrosis factor-alpha (TNFα) and NADPH oxidase subunits of 17-month-old mice. Abbreviations used are the same as in Fig. 1. * P < 0.05, †P < 0.01 between groups.

Cerebral TNF-α mRNA levels were significantly increased by high-fat diet feeding in wild-type mice but not in ASK1-/- mice (Fig. 4). As shown in Supplementary Figure 6, there was no detectable cerebral deposition of amyloid β_1−40_ or amyloid β_1−42_ in both strains of mice, irrespective of diet.

### Vascular function and blood pressure

High-fat diet significantly impaired vascular endothelium-dependent relaxation in wild-type mice but not in ASK1-/- mice (Supplementary Figure 7 (a)). Vascular endothelium-independent relaxation did not differ among 4 groups of mice (Supplementary Figure 7 (b)). There was no significant difference in blood pressure among each group of mice (Supplementary Figure 8).

### White adipose tissue adiponectin and cytokine mRNA levels

As shown in Supplementary Figure 9 (a), in both 17-week-old wild-type and ASK1-/- mice, adipose adiponectin mRNA levels were significantly lower in high-fat fed group than in control diet fed group. There was no significant difference in adipose adiponectin levels between wild-type and ASK1-/- mice fed high-fat diet. As shown in Supplementary Figure 9 (b) and (c), adipose TNF-α and IL−1β mRNA levels were significantly larger in high-fat fed ASK1-/- mice than in control diet fed ASK1-/- mice, but no significant difference was noted between high-fat fed groups of wild-type and ASK1-/- mice regarding TNF-α and IL−1β mRNA levels. No significant difference was found between two strains of mice with respect to MCP−1 mRNA (Supplementary Figure 9 (d)).

### Serum thyroid hormone and adiponectin levels

As shown in Supplementary Figure 10 (a), there was no significant difference in serum total triiodothyronine levels between the two strains of mice irrespective of diet. Serum total thyroxine concentrations were significantly lower in high-fat fed group than in control group regardless of wild-type or ASK1-/- mice, and no significant difference was noted between the two strains of mice fed high-fat diet (Supplementary Figure 10 (b)). Serum adiponectin levels were lower in high-fat diet fed group than in control diet group irrespective of the strain of mice (Supplementary Figure 10 (c)).

### Blood biochemical data

As shown in Supplementary Table 1, serum albumin was significantly lower in high-fat diet fed mice than control diet fed mice at 23 months of age, irrespective of wild-type or ASK1-/- mice. There was no difference between 23-month-old wild-type and ASK1-/- mice regarding glucose, total cholesterol, creatinine, creatinine kinase, asparatate aminotransferase, alanine aminotransferase, alkaline phosphatase, or lactate dehydrogenase, irrespective of control or high-fat diet. As shown in Supplementary Figure 11, there was no significant relationship between cognitive function and serum albumin levels (r = 0.093, p = 0.607) or serum total cholesterol levels (r = 0.126, p = 0.485).

## Discussion

Accumulating experimental evidence establish that chronic high-fat diet feeding causes obesity, insulin resistance, and glucose intolerance^8^,^9^,^10^. However, the influence of high-fat diet feeding on cognitive function is poorly understood^16^. The main purpose of the present study was to examine the influence of very long-term high-fat diet feeding on cognitive function and the role of ASK1. The major findings of this study were that very long-term high-fat diet feeding itself caused the impairment of cognitive function in mice and ASK1 was involved in this diet-induced cognitive impairment. Therefore, our present work demonstrates the novel role of high-fat diet in cognitive function and highlights ASK1 as a potential target for treatment of cognitive impairment.

**Figure 5:**
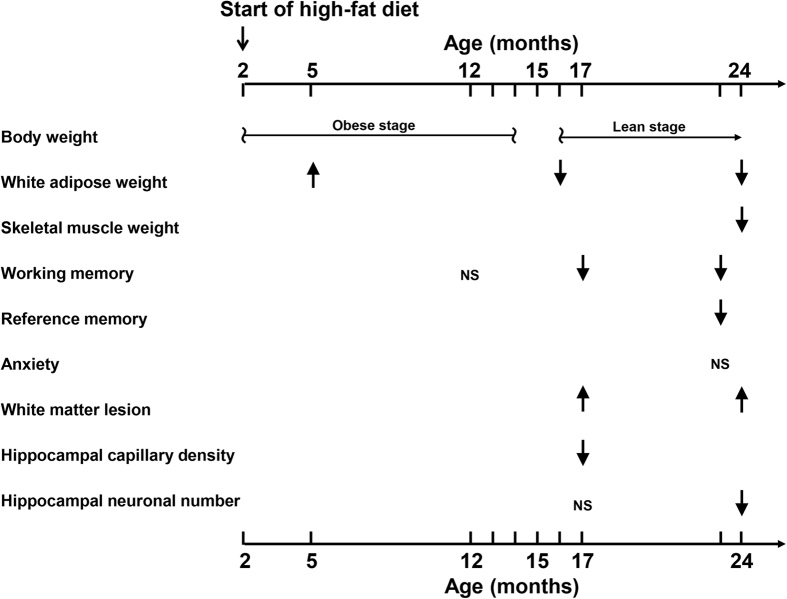
Schema summarizing the effect of high-fat diet feeding on body weight, white adipose weight, skeletal muscle weight, working or reference memory, anxiety, white matter lesion, and hippocampal capillary density and neuron number in wild-type mice. ↑ indicates increase by high-fat diet compared with control diet. ↓ indicates decrease or impairment by high-fat diet compared with control diet. NS indicates no difference between high-fat diet and control diet in wild-type mice.

Our findings on the influence of high-fat diet in wild-type mice over about 2 years are summarized in Fig. 5. In the present study, we obtained the evidence that long-term high-fat diet caused the impairment of cognitive function in wild-type mice, as characterized by deficits of spatial working memory (Y-Maze Test), and long-term memory deficits (Passive Avoidance Test). This cognitive impairment in wild-type mice was associated with white matter lesions, reduction of hippocampal capillary density, loss of hippocampal neurons, and vascular endothelial impairment, thereby supporting the notion that very long-term high-fat diet might cause cerebral hypoperfusion-induced hippocampal neuronal damage and white matter injury. Furthermore, cerebral TNF-α expression was significantly enhanced by high-fat diet, suggesting that high-fat diet might enhance cerebral inflammation. On the other hand, high-fat diet did not affect deposition of amyloid β in the brain and did not alter blood pressure in mice, suggesting that amyloid β or blood pressure might play a minor role in the development of cognitive dysfunction by high-fat diet. Of note, the elimination of high-fat-induced cognitive impairment by ASK1 deficiency was associated with the amelioration of above mentioned brain injuries such as white matter lesion, reduction of hippocampal capillary density, hippocampal neuronal loss, vascular endothelial impairment, and significant cerebral TNF-α expression. Previous reports indicate that white matter or hippocampal injury plays a causative role in the development of cognitive impairment^2^. TNF-α, a major proinflammatory cytokine, is involved in cerebral hypoperfusion-induced cognitive impairment^15^,^17^. Furthermore, we have previously shown that ASK1 deficiency caused the amelioration of cognitive impairment in mice subjected to chronic cerebral hypoperfusion, through the suppression of cerebral hypoperfusion-induced white matter lesion or TNF-α induction^15^. Therefore, collectively, ASK1 appears to be involved in cognitive impairment caused by long-term high-fat diet feeding, through cerebral hypoperfusion-induced white matter or hippocampal injury and TNF-α induction.

Another intriguing finding of the present study was that long-term high-fat diet feeding unexpectedly reduced body weight in mice and this body weight reduction was associated with the significant reduction of white adipose tissue and of skeletal muscle weight. Generally, it is well known that high-fat diet feeding causes obesity and insulin resistance^8^,^9^,^10^. Therefore, it is well established that high-fat diet-fed mice is a useful animal model of obesity. However, previous studies regarding high-fat diet fed mice have been limited to short-term high-fat diet feeding. The period of high-fat diet feeding in previous studies is a few months and is much shorter than that of high-fat diet feeding performed in the present study. To our knowledge, the present study provided the first unexpected evidence that high-fat diet feeding for a very long-term leads to leanness in contrast to the occurrence of obesity by short-term high-fat diet feeding. This unexpected reduction of body weight by long-term high-fat diet was attributed to the significant reduction of adipose tissue and of skeletal muscle weights, as shown by the significant correlation between body weight and white adipose tissue weight or skeletal muscle weight. Body weight was significantly less in long-term high-fat fed ASK1-/- mice than in their wild-type counterparts. There was no significant difference between high-fat fed ASK1-/- mice and their wild-type counterparts regarding white adipose weight, adipose cell size, and adipose adiponectin, TNF-α, IL−1β, and MCP−1 expression. On the other hand, skeletal muscle weight in high-fat fed ASK1-/- mice was significantly less than in their wild-type counterparts. These observations suggest that less body weight in high-fat fed ASK1-/- mice than in their wild-type counterparts was attributed to less weight of skeletal muscle rather than of adipose tissue. To define the mechanism underlying the leanness of mice on the long-term high-fat diet, we measured serum thyroid hormone levels in each group of mice and found no increase in thyroid hormones in high-fat fed mice. Thus, the leanness of high-fat fed mice seems not to be mediated by the dysfunction of thyroid gland. Furthermore, food intake did not differ between wild-type and ASK1-/- mice fed high-fat diet, thereby supporting that the leanness of high-fat fed mice might not be due to appetite loss. Future study such as evaluation of energy expenditure and of absorption or excretion into feces of fat is required to locate the energy imbalance of lean mice on the long-term high-fat diet.

It is an intriguing issue to determine whether cognitive dysfunction observed in long-term high-fat fed mice is associated with the leanness of these mice. In the present study, there was no significant correlation between cognitive function and body weight, white adipose weight, or skeletal muscle weight. Furthermore, no significant correlation was noted between cognitive function and serum albumin or total cholesterol. Therefore, our present findings provided no evidence for the potential contribution of leanness to cognitive impairment. However, it cannot be completely excluded that leanness might play some role in the pathophysiology of cognitive impairment in long-term high-fat fed mice, since cognitive impairment did not occur at obese stage in mice on the high-fat diet.

In conclusion, our present study provided the first evidence that long-term high-fat diet feeding itself causes cognitive impairment and ASK1 is involved in this cognitive impairment. Thus, our work highlights ASK1 as a key target molecule for treatment of cognitive impairment. Furthermore, our work provided a novel insight into the significance of high-fat diet feeding over a lifetime.

## Methods

### Animals

C57BL/6J mice were purchased from SLC Japan (Shizuoka, Japan). ASK1-/- mice were backcrossed onto the C57BL/6J background for at least 10 generations to reduce genetic variation^18^. Animals were housed in a temperature-controlled (20 ± 2 °C) and humidity-controlled (60%) room under a 12 h light/dark cycle (8:00/20:00). All experimental protocols were approved by Committee for Laboratory Animal Care and Use in Kumamoto University. All procedures were in accordance with institutional guidelines for the care and use of laboratory animals.

### Study protocol

Male ASK1-deficient (ASK1-/-) mice and control C57BL/6J wild-type mice were divided into two groups, and were fed (1) high-fat diet or (2) control diet from 2 months of age. The first cohort of mice were fed either diet until 17 months of age. The 2nd cohort of mice were fed either diet until 24 months of age. The control diet used was D12450B (3.85 kcal/g; 10 kcal% Fat (4.4 kcal% lard), 20 kcal% Protein and 70 kcal% Carbohydrate (35 kcal% sucrose and 31 kcal% corn starch)) (Research Diets, Inc. USA). The high-fat diet was D12492 (5.24 kcal/g; 60 kcal% Fat (54 kcal% lard), 20 kcal% Protein and 20 kcal% Carbohydrate)(Research Diets, Inc. USA)^8^,^19^. The content of cholesterol in lard in these diets was 0.72 mg/g. Body weight was measured every week throughout the study. Food intake was evaluated by a metabolic cage. At specified time point, cognitive function, including the Y-maze test, Passive Avoidance test, the Light and Dark Transition test, and the Rotarod test was evaluated in each group of mice. Furthermore, blood pressure was measured in each group of mice at specified time point. At the end of the study (at 17 months of age for the 1st cohort and 24 months of age for the 2nd cohort), mice were deeply anesthetized with isoflurane. After perfusion by phosphate-buffered saline, brain, white adipose tissue, and skeletal muscle were rapidly excised for the measurement of various parameters as described below.

### Y-Maze test

Spatial working memory as a cognitive function was evaluated on mice at 12, 15, and 23 months of age by the Y-maze test, as previously described^15^. Mice were placed at the end of an arm and allowed to move freely through the maze for 8-minute sessions during the dark period (7:00 to 12:00 PM). Spontaneous alterations were defined as the consecutive entry of a mouse into all three different arms to form a triplet of non-repeated components. The percentage of spontaneous alterations was calculated as the ratio of actual-to-possible alterations automatically by the Y-maze system (defined as the number of spontaneous alterations in behavior/(the total number of arm entries - 2) × 100) (Muromachi Kikai, Tokyo, Japan). The total number of arms entered during the sessions reflected locomotor activity.

### Passive avoidance test

As referencing the previous reports^20^,^21^,^22^, a cage was divided into two chambers, one of which was brightly illuminated (700 lux), and the other was dark. There was a hole between the two chambers. The floor of the dark compartment was composed of stainless steel rods connected to an electric shock generator (Muromachi Kikai, Tokyo, Japan). A mouse was placed into the light side and allowed to move freely until the mouse entered the dark side. On the learning session, when the mouse entered into the dark side, the mouse received electric shocks (1.6 mA, 3 sec) until the mouse did not enter up to a maximum of 5 minutes (Maximum three continuous sessions). On the retention session, next and third days, the time taken to enter the dark side was measured up to a maximum of 5 minutes, using a stopwatch by a well-trained operator who was blinded to the group assignments.

### Light and dark transition test

As previously described^15^, a cage was divided into two chambers, one of which was brightly illuminated (700 lux), and the other was dark. There was a hole between the two chambers. A mouse was placed into the light side and allowed to move freely for 5 minutes. A video camera was positioned over the experimental chamber, and mouse behavior was videotaped for later analysis. Each test was conducted at approximately the same time each day. The time spent in the light side were all evaluated using a stopwatch by a well-trained operator who was blinded to the group assignments.

### Rotarod test

Rotarod test was performed to assess sensorimotor coordination and balance, as previously reported^23^. The mouse was placed on the horizontal drum (Muromachi Kikai, Tokyo, Japan), and was allowed to walk forward for a maximum 60 sec to avoid the fall from the drum. The drum was started at 4 rpm and accelerated by 1 rpm every 5 sec. The time to the fall from the drum was recorded using a stopwatch. The latency was adjusted by the body weight to avoid the effects of obesity against the time to the fall from the drum.

### Assessment of white matter lesions and hippocampal neurons

Brain samples were fixed in 4% paraformaldehyde, embedded in paraffin and cut into 4 μm thick coronal sections. Sections were subjected to Klüver-Barrera staining for the measurement of white matter lesions, as previously described^15^. The severity of white matter lesions was graded as normal (Grade 0), disarrangement of the nerve fibers (Grade 1), the formation of marked vacuoles (Grade 2), and the disappearance of myelinated fibers (Grade 3).

For the hippocampal neuron counting, sections were subjected to Nissle staining. As referencing the previous report^24^, normal pyramidal cells show round and pale stained nuclei, whereas dying or dead cells show pyknotic nuclei. The numbers of surviving neurons in the CA1-hippocampal layer per 1 mm length were counted as neuronal density^24^.

### Measurements of hippocampal capillary density

To determine the change in the capillary density of hippocampus, frozen brain tissue sections were stained with the anti-CD31 antibody (rat, 1:200 dilution), reacted with horseradish peroxidase-conjugated anti-rat IgG secondary antibody, and were visualized with 3, 3’-diaminobenzidine (Dako Cytomation) for measuring the positive vessel area in the hippocampus (CA1).

### Assessment of white adipose weight and cell size

White adipose tissue weight was expressed as the sum of the epididymal, subcutaneous and mesenteric fat tissue weights. Then, epididymal adipose tissue was embedded in paraffin, was cut into 4 μm-thick sections, and were stained with hematoxylin-eosin staining. Adipocytes size was measured as adipocyte area under microscope. The cell size of at least 30 adipocytes was measured, and the average was used for the value of each sample.

### Immunohistochemistry of Amyloid β_1−40_ and β_1−42_

To determine the deposition of the Amyloid *β*_*1−40*_ *and β*_*1−42*_, frozen brain tissue sections was incubated for 30-min in 0.3% H_2_O_2_/methanol after the 5 minutes-incubation of formic acid, and then stained with A *β*_*1−40*_ and A*β*_*1−42*_ antibody (1:100, Immuno-Biological Laboratories Co., Ltd), reacted with horseradish peroxidase-conjugated anti-rabbit IgG secondary antibody, and visualized with 3, 3’-diaminobenzidine (Dako Cytomation).

### Vessel ring preparation and organ chamber experiments

As previously described^25^, thoracic aortas from mice were cut into 5 mm rings with special care to preserve the endothelium, and mounted in organ baths, filled with modified Tyrode buffer aerated with 95% O_2_ and 5% CO_2_ at 37 °C. The preparations were attached to a force transducer, and isometric tension was recorded on a polygraph. Vessel rings were primed with KCl (50 mmol/L), and then precontracted with L-phenylephrine (10^−7^ mol/L) producing a sub-maximal (70 - 80% of maximum) contraction. After the plateau was attained, the rings were exposed to increasing concentrations of acetylcholine (10^−9^ mol/L to 10^−5^ mol/L) or *S*-Nitroso-*N*-acetylpenicillamine (10^−9^ mol/L to 10^−5^ mol/L) to obtain cumulative concentration-response curves.

### Quantitative real time PCR

A detailed method was previously described^25^. We used specific oligonucleotide primers for target sequences of tumor necrosis factor-α (TNF-α)

(F 5’-AAGCCTGTAGCCCACGTCGTA-3; R 5’-GGCACCACTAGTTGGTTGTCTTTG-3’), p22 (F 5’-TGGCTACTGCTGGACGTTTCAC-3’; R 5’-CTCCAGCAGACAGATGAGCACAC-3’), gp91 (F 5’-TTGAAACCACACCTAAGCCATCTG-3’; R 5’-AACTGAGGCTTGAGACAACCTGGTA-3’), p47 (F 5’-GCGCTGGCTGGTCTATGTCA-3’; R 5’-AGGCAAATGTGGATGCTGGAA-3’), p67 (F 5’-ACTACTGCCTGACTCTGTGGTGTGA-3’; R 5’-CTGAGGCTCCGTAGTCTGCTTACTG-3’), GAPDH (F 5’-TGTGTCCGTCGTGGATCTGA -3’; R 5’-TTGCTGTTGAAGTCGCAGGAG-3’), adiponectin (F 5’-GTCAGTGGATCTGACGACACCAA-3’; R 5’-ATGCCTGCCATCCAACCTG-3’), interleukin−1β (IL−1β) (F 5’-TCCAGGATGAGGACATGAGCAC-3’; R 5’-TAACCTGCTGGTGTGTGACGTTC-3’), and monocyte chemoattractant protein−1 (MCP−1) (F 5’-GCATCCACGTGTTGGCTCA-3’; R 5’-CTCCAGCCTACTCATTGGGATC-3’).

In individual samples, each mRNA value was corrected for GAPDH mRNA value.

### Measurement of blood pressure

Blood pressure of mice was measured at 2, 5, and 16 months of age using the tail cuff method (BP-98A; Softron Co, Tokyo, Japan), as described^25^.

### Measurement of thyroid hormones, adiponectin, and other biochemical parameters in serum

Serum concentrations of total triiodothyronine and total thyroxine were measured by the electrochemiluminescence immunoassay (ECLIA). Serum adiponectin concentrations were measured by Adiponectin ELISA Kit (Otsuka Pharmaceutical Co., Ltd., Tokyo, Japan). Other biochemical parameters were measured by BioMajesy JCA-BM6050 at Kumamoto Mouse Clinic of Institute of Resource Development and Analysis, Kumamoto University.

### Statistical analysis

To determine whether the samples were normally distributed, we used the Shapiro-Wilk test. One-way ANOVA followed by Fisher’s test was used to compare the different groups. For the isometric tension studies, two-way ANOVA with repeated measures followed by Bonferroni test was used to compare the results from different groups. Depending to the normal distribution, the Pearson and Spearman correlation tests were used to analyze the correlation between body weight and other parameters. The standard Kaplan–Meier analysis was used to compare the survival rate in the groups. Data were expressed as the mean ± standard error (SEM), and p < 0.05 was considered statistically significant. All statistical analyses were carried out using IBM SPSS statistical software, version 21.0 (SPSS Inc., Chicago, IL, USA).

## Additional Information

**How to cite this article**: Toyama, K. *et al.* ASK1 is involved in cognitive impairment caused by long-term high-fat diet feeding in mice. *Sci. Rep.* **5**, 10844; doi: 10.1038/srep10844 (2015).

## Supplementary Material

Supplementary Information
