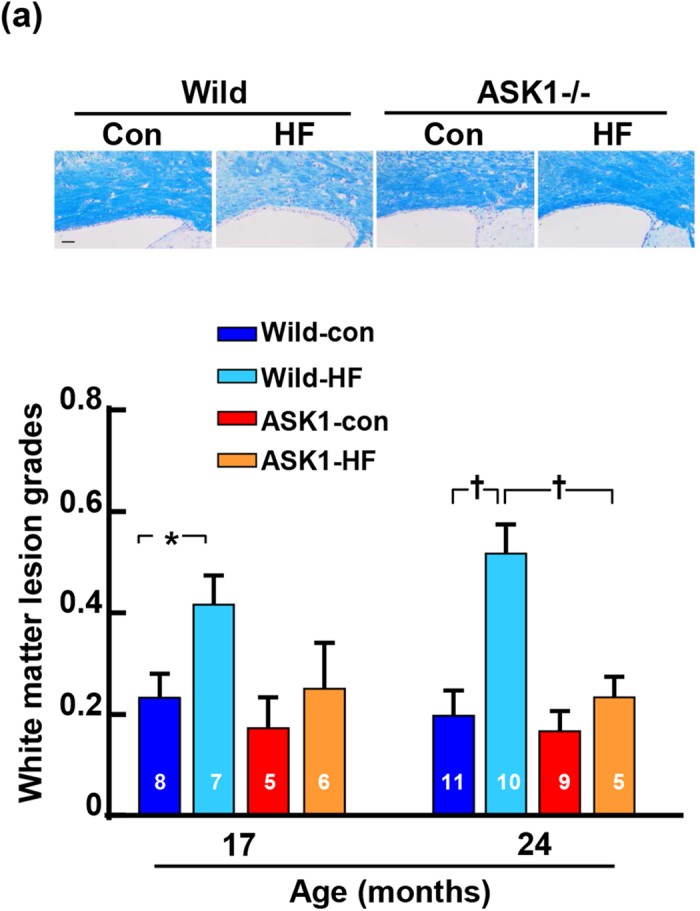# Erratum: ASK1 is involved in cognitive impairment caused by long-term high-fat diet feeding in mice

**DOI:** 10.1038/srep14028

**Published:** 2015-10-08

**Authors:** Kensuke Toyama, Nobutaka Koibuchi, Yu Hasegawa, Ken Uekawa, Osamu Yasuda, Daisuke Sueta, Takashi Nakagawa, Mingjie Ma, Hiroaki Kusaka, Bowen Lin, Hisao Ogawa, Hidenori Ichijo, Shokei Kim-Mitsuyama

Scientific Reports 5: Article number: 1084410.1038/srep10844; published online: 06052015; updated: 10082015

The original version of this Article contained a typographical error in the spelling of the author Hidenori Ichijo which was incorrectly given as Hidenor Ichijo.

In addition, there were errors in Fig. 2(a), where the number of mice in the 12 and 23 month old ASK1-HF groups should read ‘12’ and ‘7’ respectively. And in Fig. 3(a), the horizontal statistical bar should span across the Wild-HF and ASK1-HF group for the 24 month old mice. The correct Figs 2(a) and 3(a) appear below as Figs 1 and 2 respectively.

These errors have now been corrected in both the PDF and HTML versions of the Article.

## Figures and Tables

**Figure 1:**
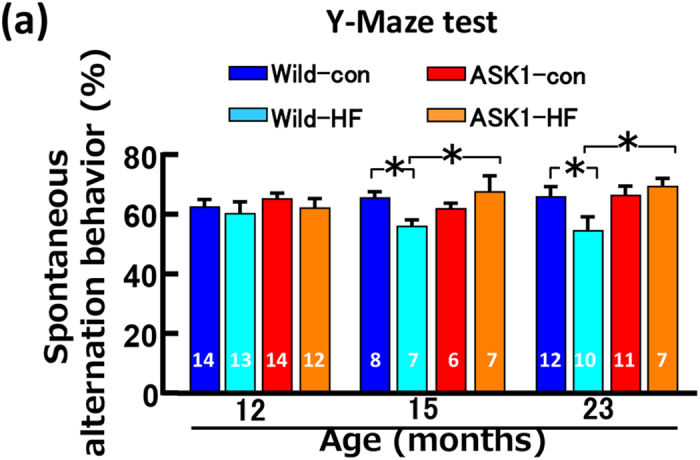


**Figure 2:**