# Supplementary material for: ASK1 is involved in cognitive impairment caused by long-term high-fat diet feeding in mice
Source: Sci Rep. 2015 Jun 5;5:10844. doi: 10.1038/srep10844 (PMC5377457; doi:10.1038/srep10844)

ASK1 is involved in cognitive impairment caused by long-term high-fat diet feeding in mice

Short title: Long-term high fat diet and ASK1

[a] **Kensuke Toyama**; [a] Nobutaka Koibuchi; [a] Yu Hasegawa; [a] Ken Uekawa; [c] Osamu Yasuda; [a] Daisuke Sueta; [a] Takashi Nakagawa; [a] Mingjie Ma; [a] Hiroaki Kusaka; [a] Bowen Lin; [b] Hisao Ogawa; [e] Hidenori Ichijo; and [a] Shokei Kim-Mitsuyama

[a] Department of Pharmacology and Molecular Therapeutics, and [b] Department of Cardiovascular Medicine, Kumamoto University Graduate School of Medical Sciences, Kumamoto, [c] Department of Cardiovascular Clinical and Translational Research, Kumamoto University Hospital, Kumamoto, [e] Laboratory of Cell Signaling, Graduate School of Pharmaceutical Sciences, and Global Center of Excellence (GCOE) program, The University of Tokyo, Japan

Supplementary Table 1. Biochemical data of blood from wild-type and ASK-/- mice fed control or high-fat diet for 21 months (from 2 to 23 months of age)

Abbreviations used: Wild-con, wild-type mice fed control diet; Wild-HF, wild-type mice fed

	Wild		ASK -/-	
	Con (n=11)	HF (n=10)	Con (n=8)	HF (n=4)
Albumin (g/dl)	0.64±0.03	0.49±0.03*	0.61±0.03	0.43±0.05‡
AST (IU/L)	128±30	147±51	101±14	179±65
ALT (IU/L)	38.8±15.5	55.1±20.0	20.9±2.9	72.8±29.2
ALP (IU/L)	149±17	112±13	136±13	166±21
LDH (IU/L)	693±196	463±37	485±39	551±65
Glucose (mg/dl)	148±13	134±12	169±14	136±16
Total cholesterol (mg/dl)	113.0±6.3	98.1±5.3	86.9±6.8	96±5.7
Creatinine (mg/dl)	0.12±0.02	0.10±0.01	0.10±0.01	0.10±0.01
Creatinine Kinase (IU/ml)	235±54	162±34	328±87	134±17

high-fat diet; ASK1-con, ASK1-/- mice fed control diet; ASK1-HF, ASK1-/- mice fed high-fat diet; AST, Asparatate Aminotransferase; ALT, Alanine Aminotransferase; ALP, Alkaline phosphatase; LDH, Lactate Dehydrogenase. The numbers in parenthesis indicate the number of animals examined. Values are the means ± SEM. #P<0.05, *P<0.01 vs Con of Wild. ‡P<0.01 vs Con of ASK -/-.

Supplementary Figure 1: Effects of long-term high-fat diet feeding on body weight at 5, 16, and 24 months of age

Abbreviations used: Wild-con, wild-type mice fed control diet; Wild-HF, wild-type mice fed high-fat diet; ASK1-con, ASK1-/- mice fed control diet; ASK1-HF, ASK1-/- mice fed high-fat diet. Values are the means ± SEM. The number within the bar indicates the number of mice examined. * P<0.05, † P<0.01 between groups.

Supplementary Figure 2: Effects of long-term high-fat diet feeding on tibia length (a), white adipose weight (b), adipose cell size (c), and relationship between body weight and white adipose weight (d)

The number within the bar in (b) indicates the number of mice examined. (a), (b), and (c) show the data obtained from 24-month-old mice. In (c), upper panels indicate representative photomicrograph of hematoxylin-eosin-stained white adipose tissue sections. Abbreviations used are the same as in Supplementary Figure 1. TL, tibia length. Scale bar = 50 μm in (c). Values are the means ± SEM. * P<0.05, † P<0.01 between groups.

Supplementary Figure 3: Effects of long-term high-fat diet feeding on musculus soleus weight (a), gastrocnemius weight (b), latency to fall (rotarod test) (c), and relationship between body weight and musculus soleus weight (d), or gastrocnemius weight (e)

(a), (b), (d), and (e) indicate the data obtained from 24-month-old mice. (c) indicates the data from 23-month-old mice. Abbreviations used are the same as in Supplementary Figure 1. Values are the means ± SEM. * P<0.05, †P<0.01 between groups.

Supplementary Figure 4: Food intake in each group of mice at 7, 12, and 16 months of age

Abbreviations used are the same as in Supplementary Figure 1. Values are the means ± SEM. †P<0.01 between groups.

Supplementary Figure 5: Relationship between cognitive function (spontaneous alteration behavior estimated by Y-Maze test) and body weight (a), white adipose tissue weight (b), or musculus soleus weight (c)

Spontaneous alteration behavior estimated by Y-Maze test, and body weight were obtained from 23-month–old mice. White adipose tissue weight and musculus soleus weight were obtained from 24-month-old mice

Supplementary Figure 6: Immunohistochemical staining with amyloid $\beta_{1\text{-}40}$ or amyloid $\beta_{1\text{-}42}$ of cerebral sections from each group of mice

Panels indicate representative photomicrographs of cerebral sections from 17-month-old mice. Abbreviations used are the same as in Supplementary Figure 1. Positive control (cerebral section from 5XFAD mice, which is a mouse model of Alzheimer's disease) showed the significant staining of amyloid $\beta_{1\text{-}40}$ and amyloid $\beta_{1\text{-}42}$. Scale bar = 100 μm

Supplementary Figure 7: Effects of long-term high fat diet on vascular endothelium-dependent relaxation with acetylcholine (a) and endothelium-independent relaxation with *S*-Nitroso-*N*-acetylpenicillamine (b)

The data was obtained from each group of 15-month-old mice.

Abbreviations used are the same as in Supplementary Figure 1. Values are the means ± SEM. * P<0.05 vs Wild-con, † P<0.05 vs ASK1-/- HF.

Supplementary Figure 8: Effects of long-term high-fat diet feeding on blood pressure

The number within the bar indicates the number of mice examined. Abbreviations used are the same as in Supplementary Figure 1. Values are the means ± SEM.

Supplementary Figure 9: Adipose tissue mRNA levels for adiponectin (a), tumor necrosis factor-α (TNF-α) (b), interleukin-1β (IL-1β) (c), and monocyte chemoattractant protein-1 (MCP-1) (d) from 17-month-old wild-type and ASK1-/- mice fed control or high-fat diet

Abbreviations used are the same as in Supplementary Figure 1. Values are the means ± SEM. The number within the bar indicates the number of mice examined. * P<0.05, † P<0.01 between groups.

Supplementary Figure 10: Serum concentrations of total triiodothyronine (a), total thyroxine (b), and adiponectin (c) from 17-month-old wild-type and ASK1-/- mice fed control or high-fat diet

Abbreviations used are the same as in Supplementary Figure 1. Values are the means ± SEM. The number within the bar indicates the number of mice examined. * P<0.05, † P<0.01 between groups.

Supplementary Figure 11: Relationship between cognitive function (spontaneous alteration behavior estimated by Y-Maze test) and serum albumin (a) or serum total cholesterol (b)

Spontaneous alteration behavior estimated by Y-Maze test was obtained from 23-month–old mice. Serum albumin and serum total cholesterol were obtained from 24-month-old mice

Supplementary Figure 1

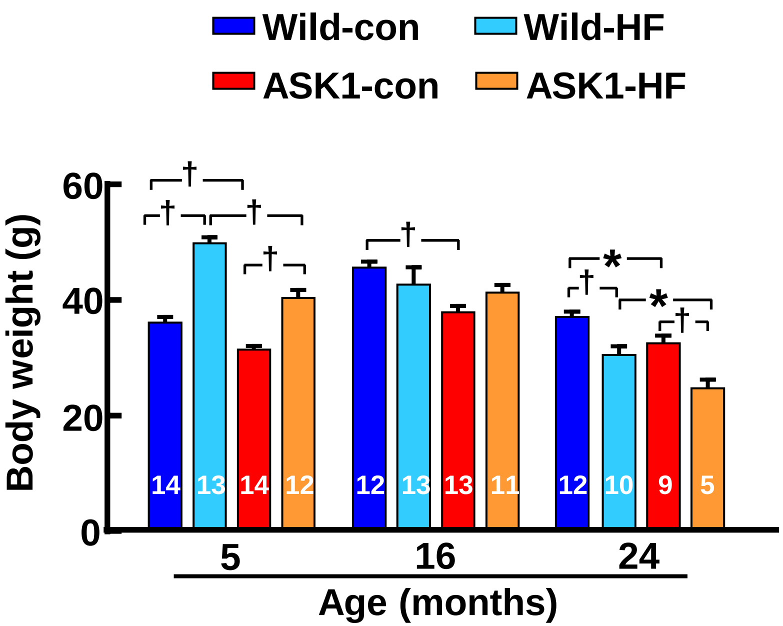

Supplementary Figure 2

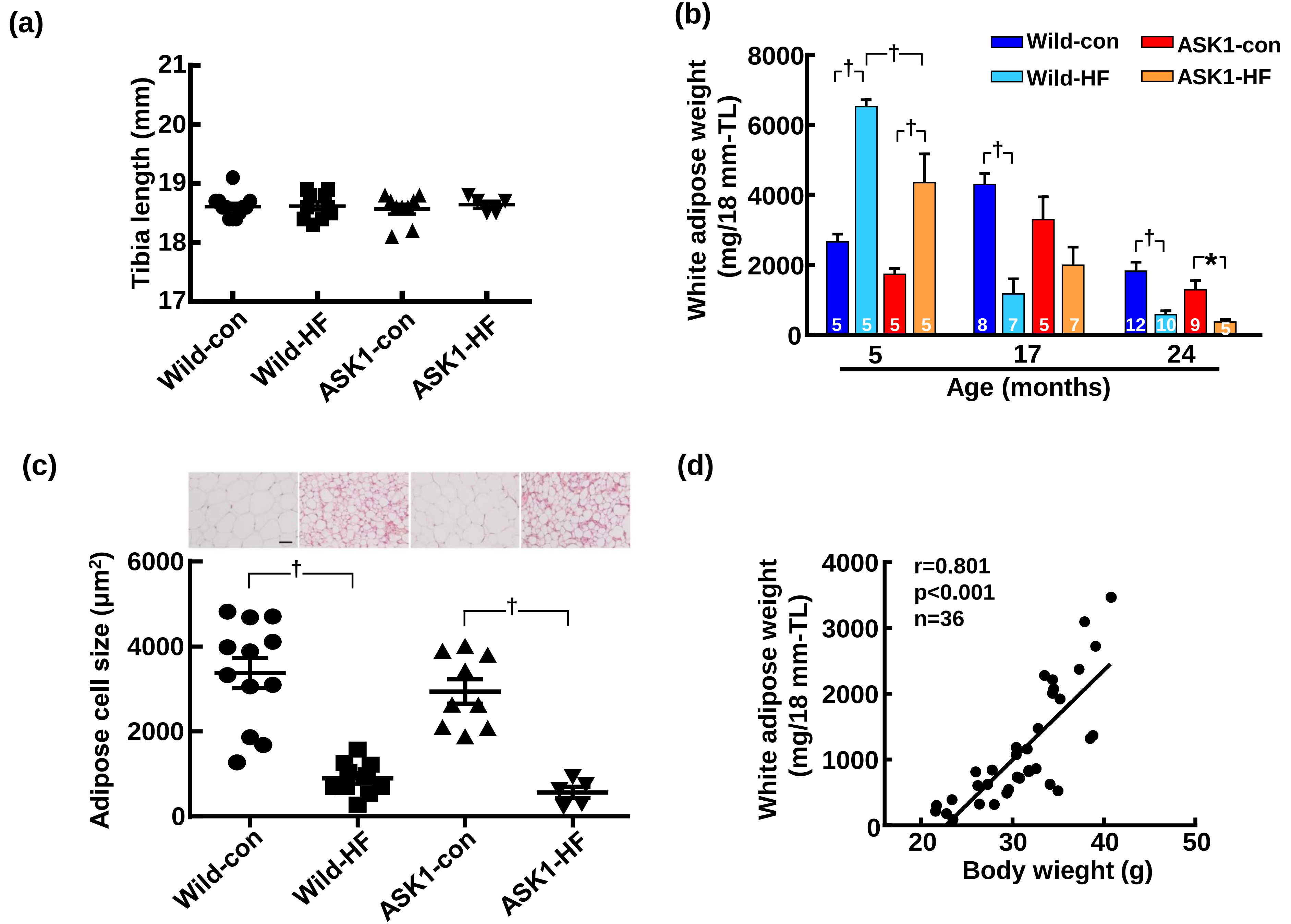

Supplementary Figure 3

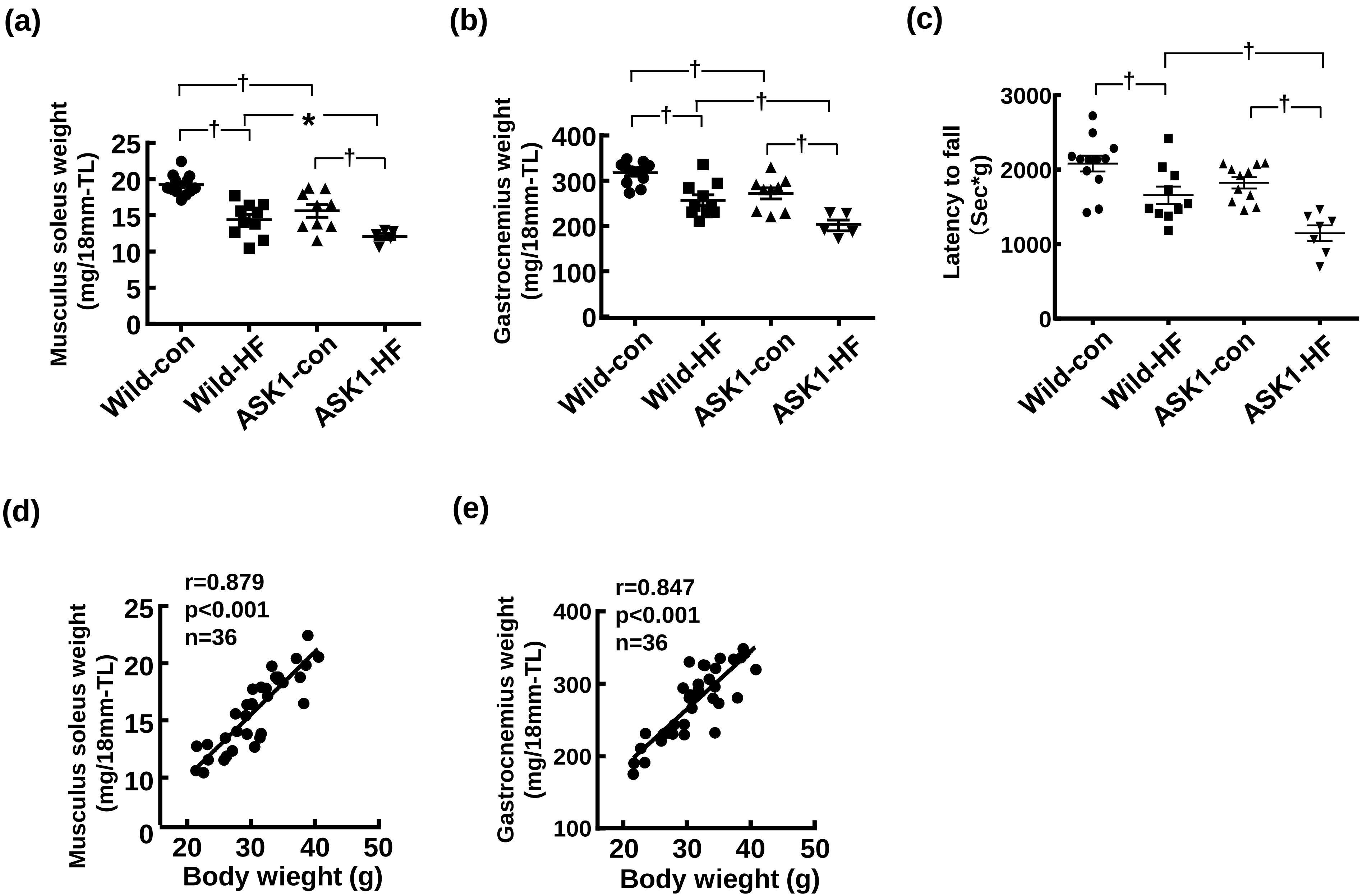

Supplementary Figure 4

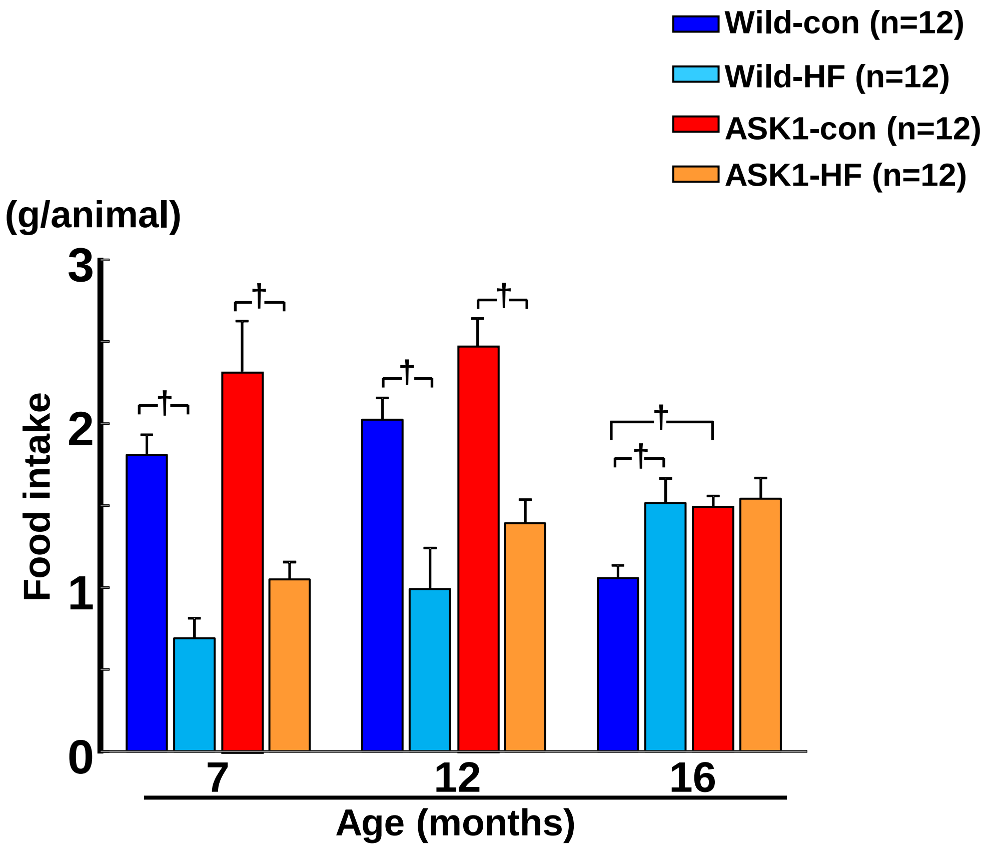

Supplementary Figure 5

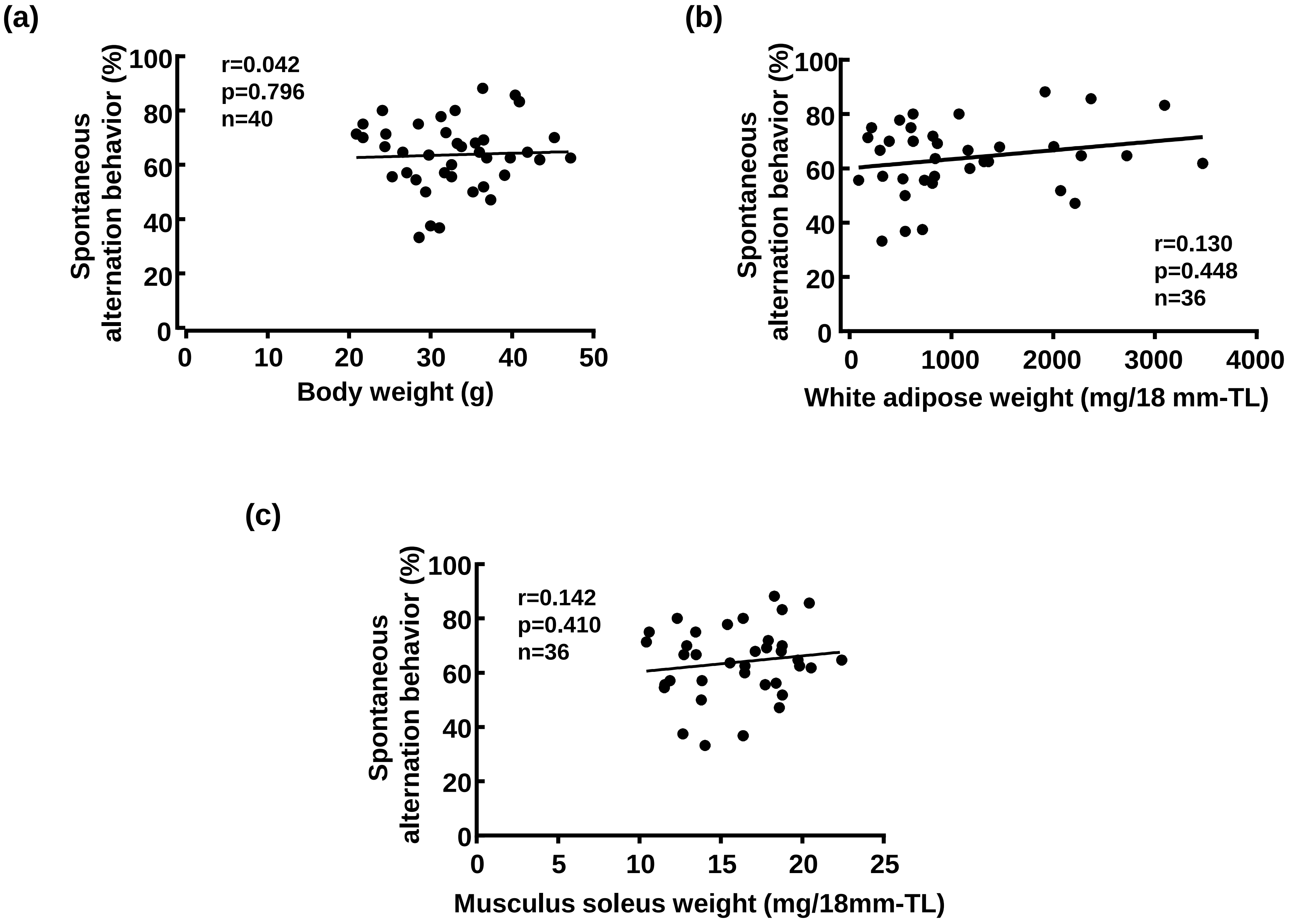

Supplementary Figure 6

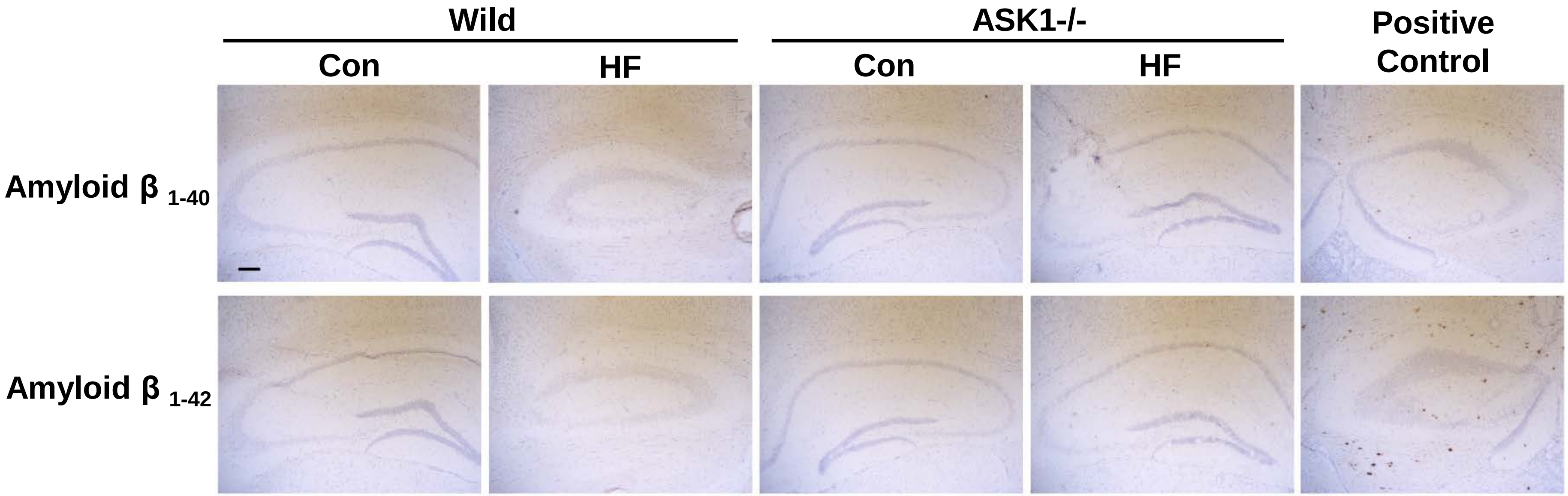

Supplementary Figure 7

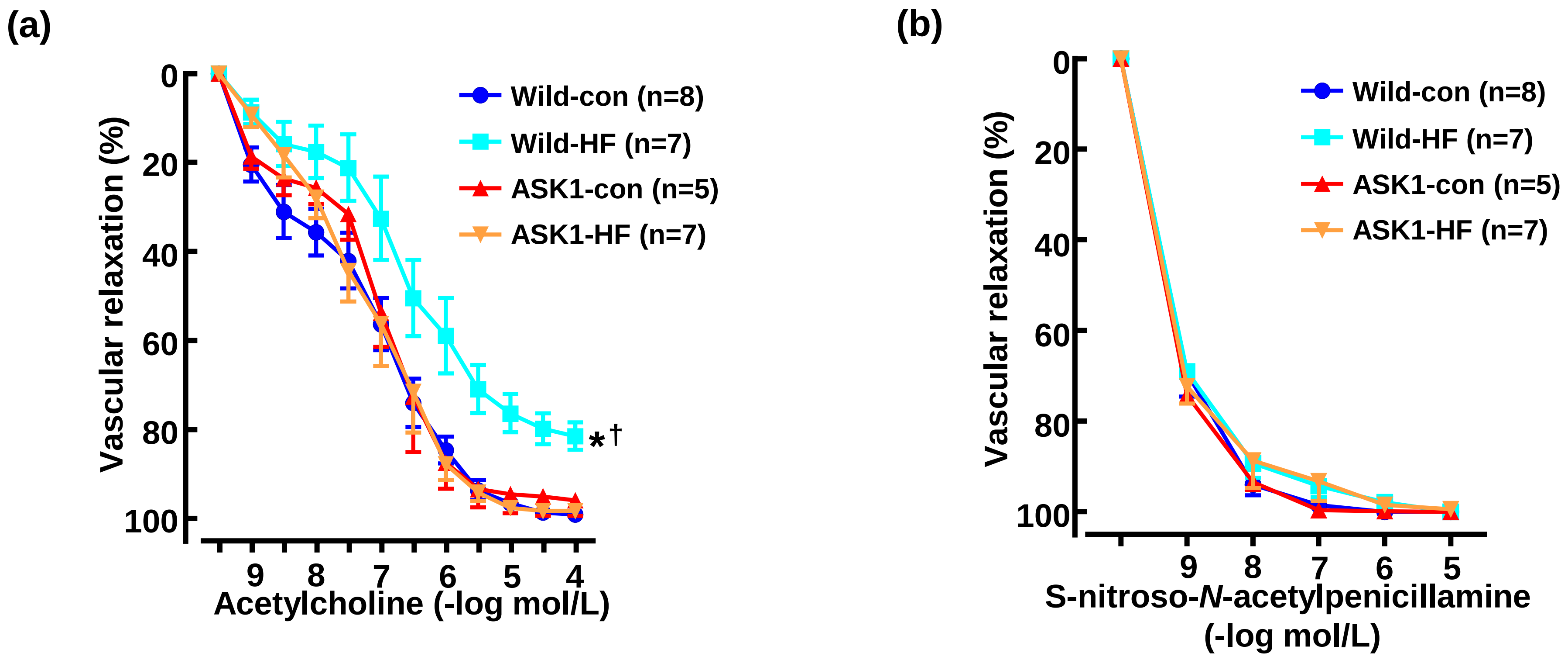

Supplementary Figure 8

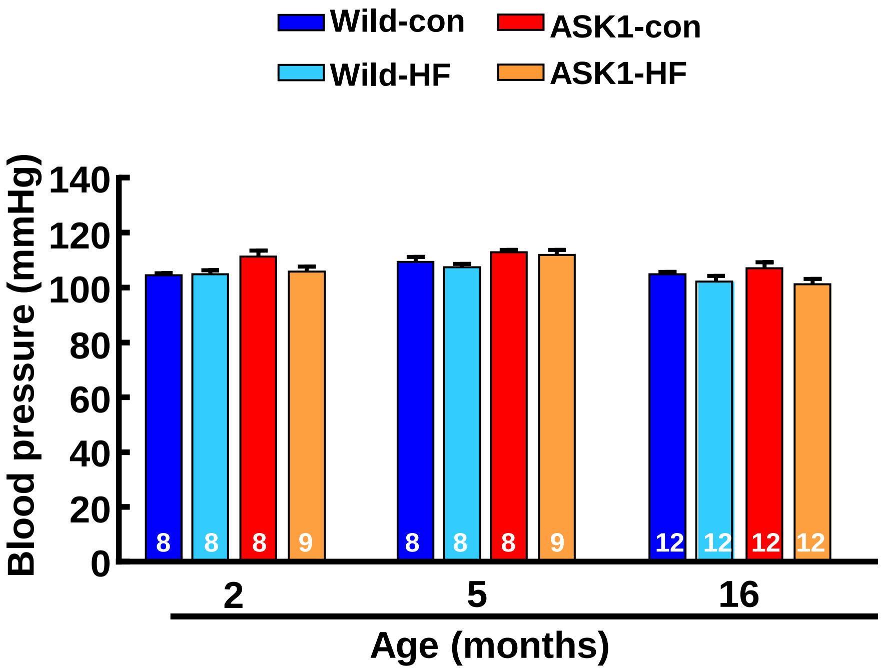

Supplementary Figure 9

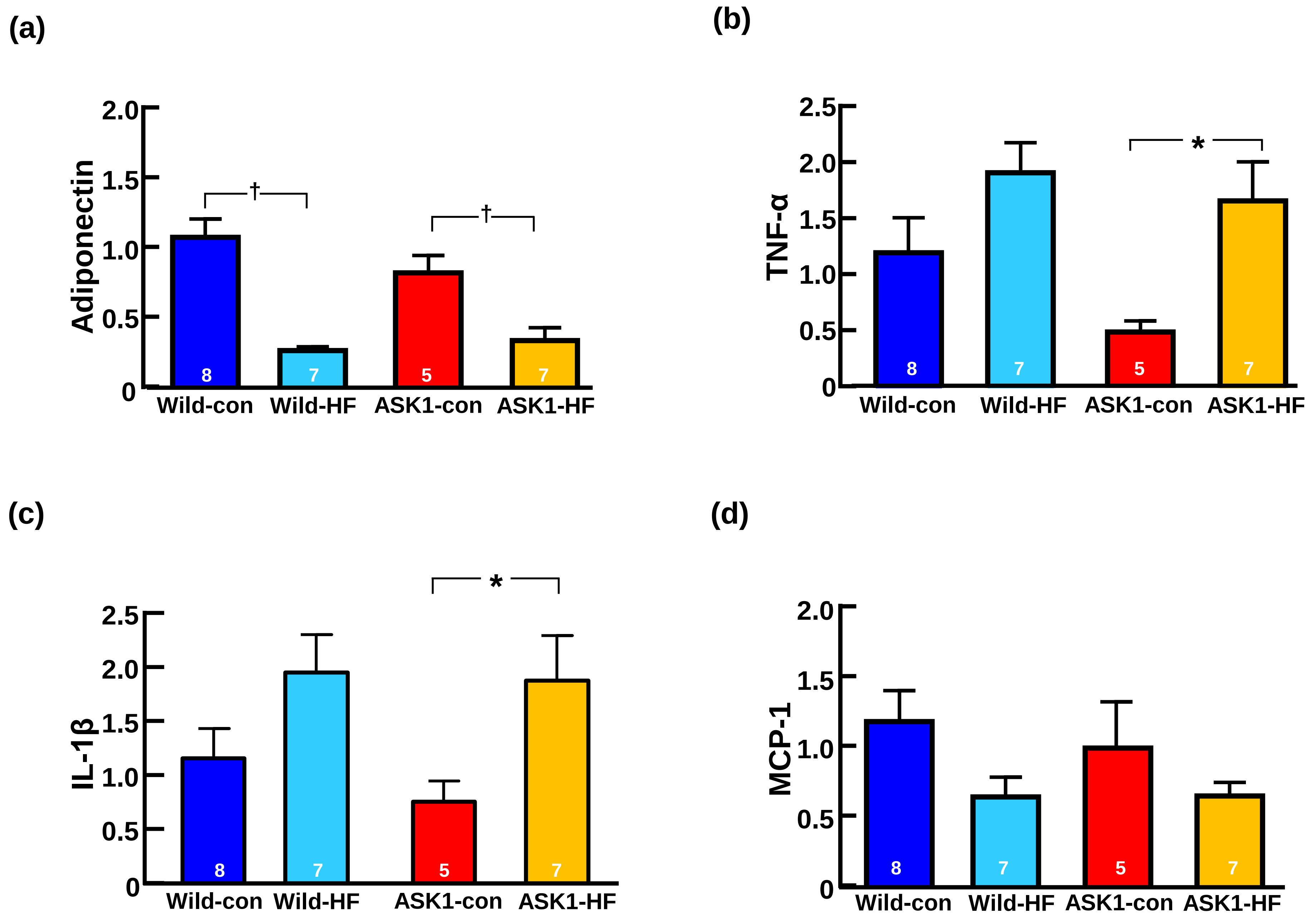

Supplementary Figure 10

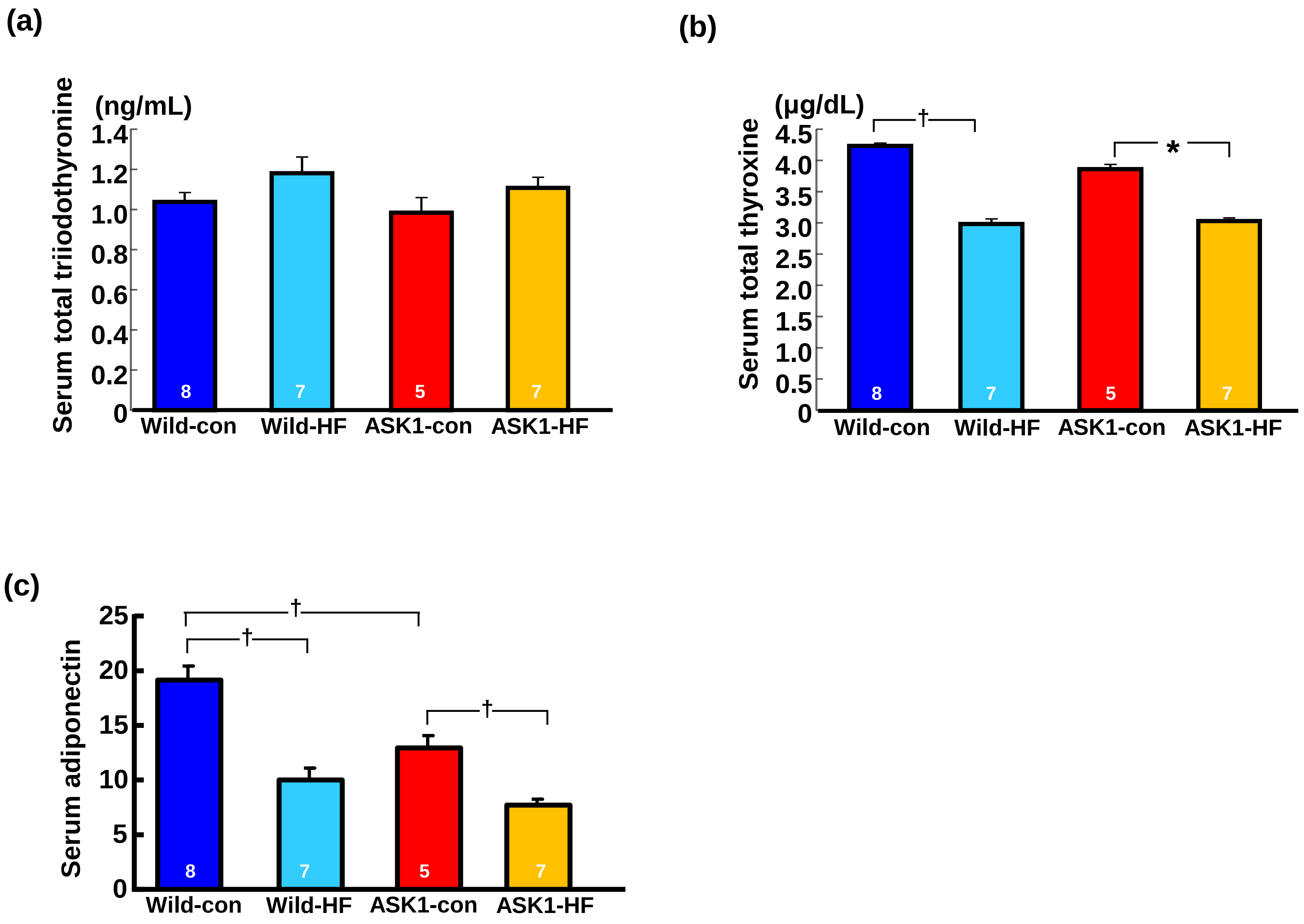

Supplementary Figure 11

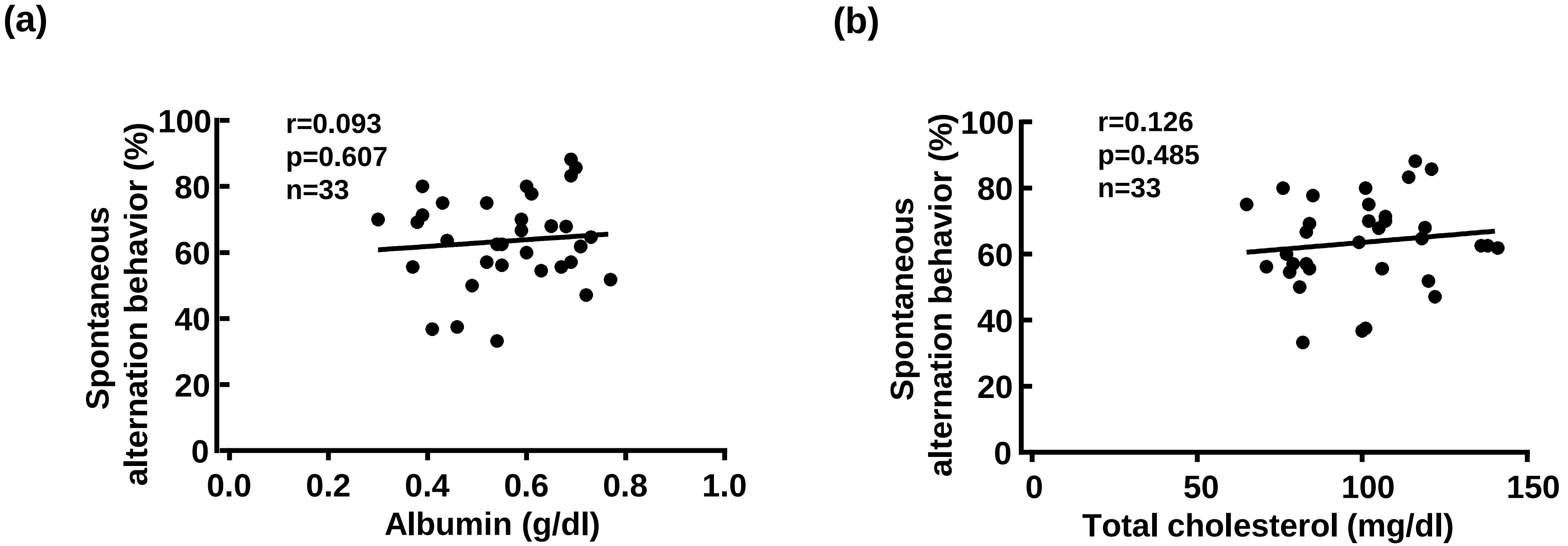

Supplement: Supplementary Information [file srep10844-s1.pdf]
